# Modulating AHR function offers exciting therapeutic potential in gut immunity and inflammation

**DOI:** 10.1186/s13578-023-01046-y

**Published:** 2023-05-13

**Authors:** Yue Chen, Yadong Wang, Yawei Fu, Yulong Yin, Kang Xu

**Affiliations:** 1 Key Laboratory of Agro-ecological Processes in Subtropical Region, Institute of Subtropical Agriculture, Chinese Academy of Sciences, Changsha, 410125 China; 2 College of Animal Science and Technology, Henan Agricultural University, Zhengzhou, 450000 China

**Keywords:** AHR, AHR ligands, Tryptophan, Gut immunity, Gut inflammation

## Abstract

Aryl hydrocarbon receptor (AHR) is a ligand-dependent transcription factor. 2,3,7,8-tetrachlorodibenzo-p-dioxin (TCDD) is a classical exogenous synthetic ligand of AHR that has significant immunotoxic effects. Activation of AHR has beneficial effects on intestinal immune responses, but inactivation or overactivation of AHR can lead to intestinal immune dysregulation and even intestinal diseases. Sustained potent activation of AHR by TCDD results in impairment of the intestinal epithelial barrier. However, currently, AHR research has been more focused on elucidating physiologic AHR function than on dioxin toxicity. The appropriate level of AHR activation plays a role in maintaining gut health and protecting against intestinal inflammation. Therefore, AHR offers a crucial target to modulate intestinal immunity and inflammation. Herein, we summarize our current understanding of the relationship between AHR and intestinal immunity, the ways in which AHR affects intestinal immunity and inflammation, the effects of AHR activity on intestinal immunity and inflammation, and the effect of dietary habits on intestinal health through AHR. Finally, we discuss the therapeutic role of AHR in maintaining gut homeostasis and relieving inflammation.

**Graphical Abstract:**

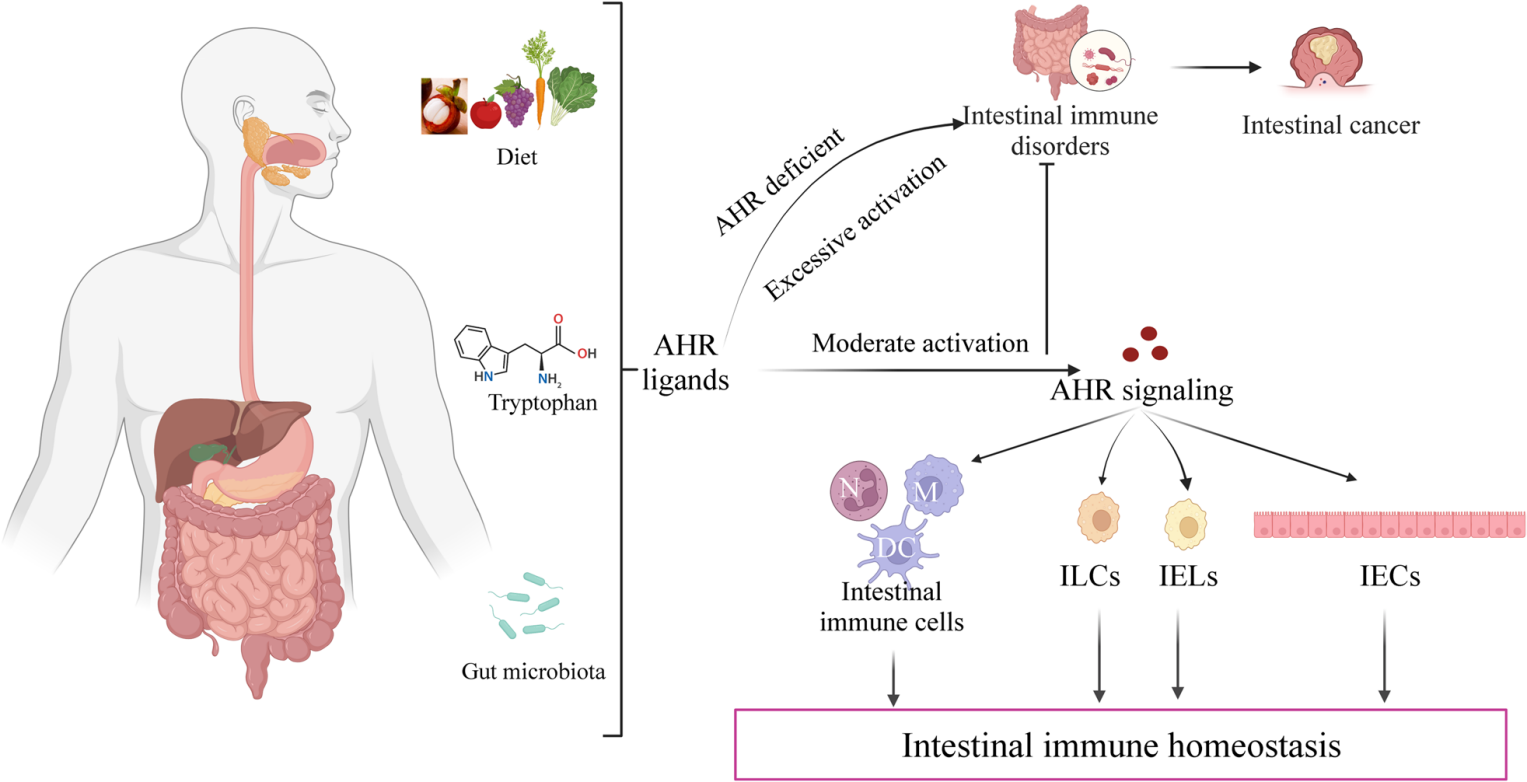

## Introduction

Aryl hydrocarbon receptor (AHR) is a critical mediator that modulates the effect of environmental stimuli, such as alterations in the circadian rhythm, oxygen tension, as well as redox potential, on organisms [1]. In the past, exogenous contaminants like 2,3,7,8-tetrachlorodibenzo-p-dioxin (TCDD) and B(a)P were considered the only ligands of AHR, and TCDD-mediated sustained AHR activation often leads to toxic results such as cell cycle arrest, chloracne and increased atherosclerosis [2–5]. However, with the discovery of more types of AHR ligands, AHR research has been more focused on elucidating physiologic AHR function than on dioxin toxicity [6].

A majority of the natural exogenous AHR ligands derived from foods and herbal medicines are beneficial for health. For instance, flavonoids are ubiquitous in many fruits and vegetables and have chemopreventive effects against colorectal cancer [7]. In addition to lowering the risk of postoperative myocardial infarction, oral curcumin also decreased the concentrations of plasma indicators for inflammation, oxidation, and damage [8]. A carotenoid-rich diet promotes healthier lives and lower chronic disease mortality [9]. The dietary metabolite indole-3-carbinol (I3C) reduces proinflammatory responses [10]. I3C and 3,3-diindolylmethane (DIM) have been shown to be effective in suppressing carcinogenesis [11–13]. Several of the most basic endogenous ligands like indolo [3,2-b] carbazole (ICZ), 6-formylindoleo[3,2-b] carbazole (FICZ)), and 6,12-diformylindolo[3,2-b] carbazole (dFICZ) bind to AHR and function as immunomodulators [14]. Kynurenine (KYN) generated in the tumor microenvironment induces AHR activation, which is related to glioma-associated immunosuppression [15, 16]. AHR activation by bilirubin suppresses experimental colitis, which indicates that bilirubin exerts an AHR-dependent anti-inflammatory effect [17]. Numerous microbial products, including tryptophan metabolites and short-chain fatty acids (SCFAs), induce AHR activation. Tryptophan metabolites are crucial for immune and inflammatory responses [18, 19]. Indole-3-aldehyde (IAld) promotes intestinal homeostasis by activating AHR [20]. SCFAs are required for the intestinal epithelium to maintain homeostasis [21].

AHR is currently considered to be a significant developmental factor and physiological regulator in the intestinal immune system. Proper activation of AHR can promote intestinal immunity and reduce the occurrence of intestinal inflammation. Under normal conditions, persistent organic pollutants alter the metabolic homeostasis of the host and the gut flora by AHR activation [22]. The proinflammatory immune response will be improved when AHR activation is minimal, whereas stronger and sustained activation of AHR disrupts intestinal flora [23]. Under inflammatory conditions, AHR activation decreases cytokine (TNF, IFNγ, IL-7, IL-12, IL-17, and IL-6) production in the intestine [24]. By changing the composition of the intestinal microbiome, AHR activation by natural ligands prevents pathogenic intestinal microbial dysbiosis [25].

Some studies have shown that AHR is crucial for maintaining intestinal health and inhibiting intestinal infections [26]. The regulatory role of AHR in intestinal inflammation has drawn increasing attention [27]. However, there is no systematic understanding of how AHR regulates intestinal immune development or influences intestinal inflammation and immune homeostasis. So, the objective of this review is to discuss the therapeutic role of AHR in maintaining gut homeostasis and relieving inflammation. Specifically, the connection between AHR and intestinal immunity, the ways in which AHR affects intestinal immunity and inflammation, and how dietary habits affect intestinal health via AHR will be discussed.

## AHR and ligands

AHR is a ligand-activated transcription factor that belongs to the basic helix-loop-helix-Per-Arnt-Sim homology superfamily (bHLH-PAS) of proteins [28, 29], which consists of a DNA binding domain, a ligand binding domain and a transactivation domain [30]. AHR has been stably expressed in animal cells for 550 million years. The high degree of conservation of AHR implicates it in a variety of physiological processes (cell differentiation, pluripotency, and stemness) [31]. In the 1970s, AHR was first discovered due to its high affinity for TCDD [32]. Studies have shown that environmental chemicals and other xenobiotics, including halogenated aromatic hydrocarbons (HAHs) and polycyclic aromatic hydrocarbons (PAHs), are the major ligands for AHR [33, 34]. With no ligand binding, AHR is found predominantly in the cytoplasm and enters the nucleus following ligand binding [35]. AHR forms a dimer with AHR nuclear transport (ARNT) and then recruits transcription factors (ERAP140, SRC-1, RIP140, etc.) in the nucleus, which in turn regulates gene expression (cytochrome P450 family, COX-2, etc.) and then modulates the immune system and cellular homeostasis of the organism [36, 37]. Several functions of AHR can be achieved by AHR ligands, including regulation of immunity, the cell cycle, cell differentiation, chemical and microbial defense.

### Exploring AHR function

**Fig. 1:**
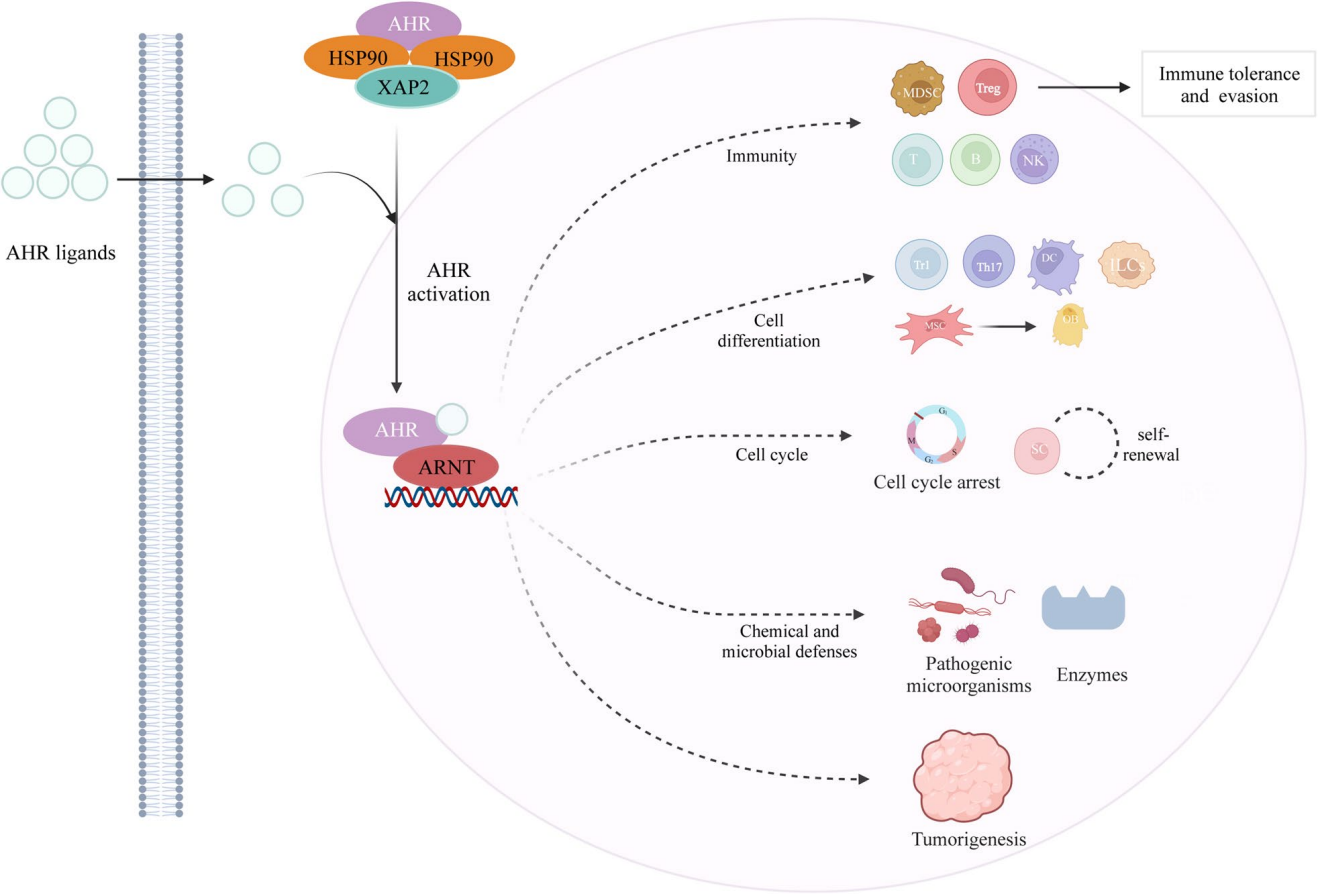
Schematic represents canonical AHR signaling and AHR function. In the absence of ligand, AHR is retained in the cytoplasm in an inactive complex containing chaperone proteins, such as HSP90 and XAP2. Upon ligand binding, AHR translocates into the nucleus, where it dimerizes with ARNT. The AhR/ARNT dimer binds genomic regions containing DRE that regulate gene expression, further exerting a variety of functions. ARNT, AHR nuclear translocator; DRE, dioxin response element; HSP90, heat shock protein 90; XAP2, HBV X-associated protein 2; MDSC, myeloid-derived suppressor cells; Treg, regulatory T cell; T, T cells; B, B cells; NK, natural killer cell; Tr1, Type 1 regulatory T; Th17, T helper cell 17; DC, dendritic cells; ILCs, innate lymphoid cells; SC, stem cell

According to the currently available evidence, AHR is crucial for immunity, the cell cycle, cell differentiation, chemical and microbial defense, and tumorigenesis [38, 39] (Fig. 1).

AHR is vital for the regulation of both innate and adaptive immune cells [40]. AHR regulates the secretion of cytokines from subcellular populations of innate and adaptive immune cells [41, 42]. The expression of AHR in the adaptive immune system is high in Th17 cells and the IL-17/IL-22 subset but low in naive T and B cells [43–45]. Furthermore, AHR regulates immune cell differentiation, such as ILCs, Th17, Treg, and Tr1 cells. AHR activation promotes immune tolerance and immune evasion by generating suppressive immune cells (MDSCs and Tregs) [46].

AHR is critical to cell cycle regulation. Low levels of AHR activity are essential for the cell cycle and stem cell self-renewal [47]. Nevertheless, AHR activation at high levels causes cell cycle arrest in the G1/G0 phase, providing conditions for cell differentiation [48]. Furthermore, AHR is crucial for cell differentiation. AHR regulates the differentiation of dendritic cells (DCs) [49]. AHR activation might promote the differentiation of mesenchymal stem cells toward osteoblasts rather than adipocytes [50]. In addition, activation of AHR influences the expression of numerous genes associated with keratinocyte differentiation [51].

AHR is essential in defending against pathogenic microorganisms through chemical and microbial defenses. AHR induces the expression of gene batteries involved in the three phases of drug metabolism, also referred to as the chemical defense, including phase I enzymes (primarily CYPs), phase II conjugate enzymes (UGTs, SULTs, and GSTs), and phase III conjugate transporters (ABCG2) [52–54]. Also, AHR is essential for defense against acute and chronic bacterial infections [55]. Studies have demonstrated that AHR-deficient mice are more susceptible to bacterial infection than wild-type mice [56].

AHR is also critical for tumorigenesis, encompassing both pro- and anti-tumorigenic activities. The function of the ligands (AHR agonist or antagonist), tumor type and the cellular and protein environment all influence the relationship between AHR and tumor [57]. Activation of AHR contributes to tumor initiation through genotoxicity [58]. Formate derived from the microbiome promotes colorectal cancer tumor invasion by activating AHR signaling [59]. Chemical exposure and AHR activation affect mammary gland differentiation processes, increasing the risk of breast cancer [58, 60]. However, the expression of AHR in IECs is a necessary condition for the reduction of premalignant lesions of the colon [61]. AHR agonists such as I3C were used as a chemopreventive therapy for IBD-associated colorectal cancer [62]. In mice, the AHR agonist FICZ inhibited SREBP2 posttranslationally and reversed tumorigenesis [63]. Furthermore, treatment with TCDD blocks the proliferation in human colorectal cancer cells [64, 65].

### AHR ligands

AHR ligands are characterized as hydrophobic molecules with aromatic rings [66]. According to their structural characteristics, they are mainly PAHs, HAHs, tryptophan derivatives, indoles and polyphenols [36]. Furthermore, AHR ligands can be classified as exogenous synthetic, natural exogenous, or endogenous ligands (Table 1).

**Table 1:** AHR ligands. A list of common AHR ligands, their abbreviations, whether they are exogenous or endogenous, whether they are synthetic or natural products, and their class and source

Type	Ligand	Abbreviation	Class	Source	References
Exogenous synthetic ligands	2,3,7,8-Tetrachlorodibenzo-p-dioxin	TCDD	Agonists	Municipal and industrial waste incineration, vehicle exhaust, PVC plastics, pesticide production, and steel smelting	Kerkvliet N. I. 2012[328]
	Benzo[a]pyrene	BaP			Park R et al. 2020; Großkopf, H et al. 2021 [329, 330]
	Benz(a)anthracene	BA			GMachala M et al. 2001 [331]
	Polychlorinated biphenyls	PCBs			Safe S. H et al. 2001[332]
	4-(3-Chloro-phenyl)-pyrimidin-2-yl) -(4-trifluoromethyl-phenyl)-amine	VAF347			Ibabao C. N et al. 2015; Zapadka T. E et al. 2021 [333, 334]
Natural exogenous ligands	Berberine	Berberine	Agonists	Dietary	Vrzal R et al. 2005 [95]
	2-(indole-3-methane)-3, 3'-diindolylmethane	LTr-1			Lin L et al. 2022 [335]
	3, 3-diindolylmethane	DIM			Chen I et al. 1996 [336]
	Indolo[3,2-b] carbazole	ICZ			Esser C et al. 2015[42]
	Flavonoids	Flavonoids	Agonists/antagonists		Xue Z et al. 2017 [93]
	Indole-3-carbinol	I3C	Weak agonists/partial antagonists		Peng C et al. 2021 [337]
	Carotenoids	Carotenoids			Zhang, S et al,.2003 [79]
	Curcumin	Curcumin	Agonists/antagonists		Ciolino H. P et al. 1998 [338]
	Resveratrol	Resveratrol			Nguyen N. T et al. 2015 [339]
Endogenous ligands	Lipoxin A4	Lipoxin A4	Agonists	Host metabolism	Denison M. S et al. 2003 [340]
	Bilirubin	Bilirubin			Bradshaw T. D et al. 2009 [141]
	Biliverdin	Biliverdin			Xue J et al. 2012[341]
	Heme metabolites	Heme metabolites			Tan Y. Q et al. 2022 [146]
	6-Formylindolo[3,2-b] carbazole	FICZ		Photo-oxidation	Smirnova A et al. 2016; Quintana F. J et al. 2008 [146, 217, 343]
	2-(10H-indole-30-carbonyl)-thiazole-4carboxylic acid methyl ester	ITE		Endogenous/chemical process	Dolciami D et al. 2018 [343]
	Kynurenine	KYN		Host metabolism	Gargaro M et al. 2021[344]
	Cinnabarinic acid	CA			Lowe M. M et al. 2014 [345]
	Kynurenic acid	KA			Novikov O et al. 2016 [346]
	Xanthurenic acid	XA			DiNatale B. C et al. 2010 [347]
	Tryptamine	Tryptamine		Microbiota metabolism	Dopkins N et al. 2021 [348]
	Skatole	skatole			Kurata K et al. 2019 [349]
	Indoleacrylic acid	IA			Lavelle A et al. 2020 [350]
	Indole-3-acid-acetic	IAA			Zhao H et al. 2019 [351]
	Indole-3-propionic acid	IPA			Lavelle A et al. 2020 [350]
	Indole-3-lactic acid	ILA			Wong C. B et al. 2020 [352]
	Indole-3-aldehyde	IAld			Zelante T et al. 2013 [20]
	Indole-3-carboxaldehyde	3-IAld			Puccetti M et al. 2021 [353]
	Indole-3-acetaldehyde	IAAld			Aoki R et al. 2018 [354]

#### Exogenous synthetic ligands

Exogenous ligands contain synthetic and natural ligands. Exogenous synthetic ligands are mainly derived from municipal and industrial waste incineration, vehicle exhaust, PVC plastics, pesticide production, and steel smelting [67, 68]. These pollutants have significant immunotoxic effects, causing a decrease in humoral and cellular immunity and inducing cancers [69]. Exogenous synthetic ligands mainly include HAHs (dioxins, furans, PCBs) and PAHs (B(a)P, BA) and their congeners [70]. TCDD binds to the AHR protein, and the TCDD/AHR/ARNT heterodimer binds to dioxin response elements in the target genes' regulatory regions, including those encoding dioxin biodegradation enzymes (CYP1A1, CYP1A2, CYP1B1). The increased expression of *CYP1A1* is a molecular marker of TCDD action [71]. 2-(4-Amino-3-methylphenyl)-5-fluorobenzothiazole (5F 203) is a high affinity ligand for AHR that antagonizes the induction of *CYP1A1* RNA by TCDD [72]. Coplanar PCBs are high affinity AHR ligands. When exposed to PCBs, IEC line monolayers increase permeability and alter junctional protein regulation [73]. PCB 153 causes DNA damage and is genotoxic to IECs. Oral exposure to PCB 153 increased IL-6 expression (15.5-fold, P < 0.01) in proximal small intestine IECs, the driver of this inflammation and increase in permeability is the transcription factor NF-kB, which can become activated through ataxia telangiectasia mutated (ATM) and the NF-KB essential modulator (NEMO) [74]. Benzo(a)pyrene (BaP), an AHR ligand, is one of the components of cigarette smoke. BaP promotes gastric cancer cell proliferation and metastasis via the AHR and ERK signaling pathways [75]. Benz(a)anthracene (BA), as a ligand, has a high binding affinity toward AHR and stimulates downstream signaling cascades that regulate tyrosinase activity and melanin synthesis [76]. VAF347, a small molecular weight compound, binds AHR, induces AHR-driven signal transduction and *CYP1A1* expression and exerts anti-inflammatory activity in monocytes [77, 78]. Overall, the exogenous synthetic ligands are the most characteristic high-affinity AHR ligands and AHR agonists.

#### Natural exogenous ligands

Many natural compounds, such as glucobrassicins, flavonoids, resveratrol, carotenoids, curcumin and berberine, are AHR agonists or partial antagonists [79]. Their anti-inflammatory properties can be explained by the activation or antagonism of AHR. Cruciferous vegetables (broccoli, Brussels sprouts, etc.) contain glucobrassicins, which upon digestion, release I3C, which is a weak AHR agonist [80]. In the stomach, I3C is converted into DIM, ICZ and 2-(indole-3-methane)-3,3'-diindolylmethane (LTr-1), which are AHR agonists. Compared with other natural products, ICZ has a higher affinity toward AHR [6]. Taken together, I3C, LTr-1, DIM and ICZ are natural exogenous AHR ligands.

Dietary flavonoids can act as AHR ligands sourced from tea, fruits, wine, vegetables and cacao, including quercetin, kaempferol and baicalin [81]. Dietary quercetin and kaempferol are AHR ligands that affect *CYP1A1* transcription and inhibit TCDD-induced AHR/DRE-driven transactivation. Moreover, quercetin is an AHR agonist that is activated indirectly by inhibiting the degradation of FICZ [82–84]. *Scutellariae Radix* inhibits AHR activity, and baicalin, one of its major active ingredients, effectively blocks the activation of AHR stimulated by cigarette smoke [85]. In addition, baicalin reduces AHR expression, which inhibits the inflammation response to myocardial ischemic injury [85]. Resveratrol, a partial agonist of AHR, induces the expression of *CYP1A1* [86]. Resveratrol has anti-inflammatory and antitumor effects by activating AHR [87]. In weaned piglets, dietary supplementation with resveratrol activates AHR and increases *CYP1A1* gene expression in the jejunal mucosa [88].

In addition, AHR ligands have been found in other dietary products, such as carotenoids, curcumin and berberine [89]. Carotenoids, as novel antagonists of AHR, are able to affect the AHR signaling pathway, probably through oxidative conversion to retinoids in the body [90]. Retinoids regulate the AHR signaling pathway not only by binding to AHR ligands, but also by regulating the crosstalk between AHR and RAR/RXR signaling [91]. The primary component of turmeric, curcumin, is an agonist and antagonist of AHR and can inhibit the conversion of AHR through its phosphorylation. In the nucleus, curcumin stimulates AHR heterodimerization with ARNT but is unable to induce AHR binding to DRE or CYP1A1 protein expression [92]. Curcumin attenuated AHR signaling by inhibiting AHR-ARNT heterodimerization, which is required for AHR transactivation [89]. Curcumin inhibits TCDD-induced DNA-binding activity of the AHR/ARNT heterodimer [92]. Curcumin reduce AHR nuclear translocation, which is challenged by AHR-agonistic B[a]P [93]. In addition, curcumin may promote ARNT proteasomal degradation by increasing intracellular oxidative stress [94]. Berberine, a quaternary isoquinoline alkaloid found in plants, activates AHR at high concentrations and for short periods [95]. Dietary AHR ligand exposure is common and constant for animals and people, which is beneficial for maintaining intestinal homeostasis under normal conditions.

#### Endogenous ligands

In addition to exogenous ligands, many endogenous compounds can activate AHR in vivo, such as heme metabolites, arachidonic acid metabolites, tryptophan metabolites, equilenin, indigo and indirubin [18, 96]. Heme metabolites like bilirubin and biliverdin directly activate AHR to induce *CYP1A1* gene transcription. Bilirubin-mediated AHR activation is associated with anti-inflammatory signaling [97]. Heme metabolites have comparatively weak affinity toward AHR when compared to TCDD [98]. Arachidonic acid metabolites like lipoxin A4 are competitive substrates for *CYP1A1* [99]. Tryptophan metabolites, including ITE, FICZ, kynurenine, cinnabarinic acid (CA), kynurenic acid (KA), and xanthurenic acid (XA), are also endogenous ligands [100–102].

The AHR agonist 2-(10H-indole-30-carbonyl)-thiazole-4carboxylic acid methyl ester (ITE) was first isolated from porcine lung [103]. ITE could significantly activate the AHR of placental trophoblast cells, induce the expression of the downstream gene *CYP1A1*, and inhibit placental trophoblast cell proliferation [104]. ITE binds to the same AHR site as TCDD, and they compete for binding but with only one percent of the affinity of TCDD [105]. Moreover, FICZ, a naturally produced photo-oxidation product of tryptophan, is another high-affinity endogenous agonist of AHR. And a small amount of FICZ is enough to activate AHR [106, 107]. FICZ has been shown to be a superior substrate for enzymes encoded by AHR-regulated genes [108]. AHR ligands, including Kynurenine, KA, XA, and CA, can induce the expression of AHR-dependent gene [109].

Bacterial tryptophan catabolites, including tryptamine, 3-methylindole (skatole), indoleacrylic acid (IA), indole-3-acid-acetic (IAA), indole-3-lactic acid (ILA), IAld, indole-3-acetaldehyde (IAAld), indole-3-carboxaldehyde (3-IAld), and indole-3-propionic acid (IPA), are also endogenous ligands of AHR in the body [20, 110–114]. AHR is activated by tryptamine to regulate intestinal immunity. On the contrary, when intestinal homeostasis is disrupted, AHR regulates tryptamine production [115]. Indigo and indirubin are endogenous AHR activators. In contrast to TCDD, indigo is an equivalent activator of AHR and indirubin is even more potent [116–118]. Endogenous ligands, commonly known as AHR agonists, are synthesized in the organism and derive from endogenous/chemical process, photo-oxidation, host metabolism and microbiota metabolism.

#### AHR is utilized and activated by ligands

AHR was first discovered during research on TCDD. As a high-affinity ligand, TCDD can lead to persistent AHR activation [119]. With industrial advancement, various exogenous AHR ligands have been synthesized, including PAHs, polybrominated dibenzo-p-dioxins, polybrominated diphenyl ethers, and other major classes of known and unknown toxicant compounds [120]. Extractable organic matter from PM_2.5_, lipophilic components of diesel exhaust extract and cigarette smoke extract all have negative impacts on organisms by activating AHR [121–124].

However, AHR is not a “dioxin receptor” but rather a factor in the maintenance of individual development and normal immune function [125]. Before these exogenous substances were synthesized, AHR was stably expressed in animal cells for 550 million years, and its high degree of conservation is consistent with AHR having essential roles in physiological and toxicological processes [126]. Hence, we speculated that AHR was being utilized by exogenous synthetic ligands, not acting as a dioxin receptor. In addition to toxic effects, TCDD was proven to have a positive impact on organisms by activating AHR, such as relieving colitis symptoms, decreasing the viral load and the levels of proinflammatory cytokines, and suppressing experimental autoimmune encephalitis [14, 127, 128].

Why does AHR have a positive effect on organisms in response to exogenous synthetic ligands? One explanation is that the AHR signaling pathway might alleviate the toxicity of exogenous toxic substances in the body by inducing their metabolism or inducing immunoreactions [129]. When exposed to exogenous toxic ligands, AHR activation induces host immunity and maintains the steady state of the organism [130]. Moreover, TCDD and some exogenous toxicants have strong oncogenic effects [131]. AHR has been identified as a tumor-associated protein and therapeutic target molecule in recent years [57]. Thus, AHR has the dual role of both suppressing toxic reactions and acting as a pattern recognition receptor to detect these risk-associated molecules and induce defenses against their general toxic effects on the body.

How can the activation of AHR be regulated? AHR activity can be regulated in multiple ways. Treatment with AHR ligands is the traditional approach for activating AHR [132]. This results in the formation of the AHR/ARNT complex, which then mediates the transcriptional activation of several genes encoding drug-metabolizing enzymes, most notably *CYP1A1* and *CYP1B1* [133]. Thus, the effect of the amount of ligand on AHR may be easier to understand. Aside from the amount of available AHR ligands, the characteristics of AHR ligands can influence the activity of AHR and further modulate intestinal immunity. The characteristics of AHR ligands can be defined as the following two main aspects: (1) Activity of the ligand in binding to AHR. (2) Its properties of being metabolized by the organism.

1. Activity of the ligand in binding to AHR

Different types of ligands have distinct effects on AHR activation. Exogenous ligands such as TCDD, BaP and other environmental pollutants have a high affinity for AHR, which can activate AHR constantly [134]. There are significant differences in activation properties among endogenous ligands, such as FICZ and ITE, which have a low content in the body but have a high affinity for AHR. However, some other endogenous ligands, such as KYNA and L-KYN, are weak agonists of AHR [100, 135]. Chewing cruciferous vegetables like broccoli and Brussels sprouts promotes the enzymatic cleavage of glucosinolates by myrosinase, producing I3C and indole-3-acetonitrile (I3ACN), which both have the ability to bind and activate AHR, albeit weakly. However, ICZ, as a derivative of I3C, potently binds and activates AHR in a manner comparable to TCDD [136]. Endogenous ligands are molecules necessary for physiological activity, and there is a negative feedback regulatory system controlling their concentration in the body [137]. Dietary-derived ligands serve important physiological functions, although they are rapidly metabolized in vivo [18]. Most dietary-derived ligands are weak AHR agonists and activate AHR at low concentrations, but at high concentrations, they act as AHR antagonists [138]. In addition, dietary-derived ligands have more significant agonistic or antagonistic effects when interacting with high-affinity ligands such as TCDD and FICZ [80].

(2) The properties of being metabolized by the organism

The properties of ligands been metabolized by the organism also influence the activity of AHR [89]. Some AHR ligands are difficult for the body to metabolize, such as TCDD and PCBs. The half-life of dioxin is about 7 years, so TCDD is exceptionally stable in biological systems [139]. Polychlorinated biphenyls (PCBs) are chlorinated compounds that are hydrophobic and lipophilic. This compound is relatively stable, difficult to degrade, accumulates in organisms for a long time, and widely exists in the environment [140]. The body can metabolize natural exogenous ligands more quickly than TCDD. After oral administration, the half-lives of quercetin, curcumin and resveratrol are 11–28 h, 28.1 h, and 9.2 h, respectively. The half-lives of I3C and DIM are 12–24 h [141–145].

Most endogenous ligands can be metabolized by organisms, such as arachidonic acid, heme, tryptophan, and other molecules [146]. Lipoxin A4 (LXA4) is a metabolite of arachidonic acid, which has no aromatic ring and complete planar structure. The metabolism of LXA4 is autoregulated by activated AHR to regulate *CYP1A1* expression [99]. Tryptophan can be catabolized through a variety of pathways in vivo, such as KYN and 5-HT [147, 148]. Tryptophan can also be converted by the gut microbiota into a variety of AHR ligands, including indole and its derivatives [96]. Thus, the activation effect of these ligands is not continuous. Their metabolic rate and the metabolites of the ligands determine the AHR activation. Dietary-derived ligands are also rapidly metabolized in vivo. Strikingly, AHR-regulated cytochrome P450 enzymes are capable of efficiently metabolizing ITE, FICZ, I3C, and curcumin [149–151].

Additionally, AHR activity can be modulated independently of ligands. For instance, in the absence of ligands, AHR may undergo nucleocytoplasmic shuttling, causing the activation of its target gene *CYP1A1* [152, 153]. Sulindac directly binds AHR and stimulates AHR: ARNT heterodimerization to drive *CYP1A1* expression [154]. Both sunitinib, a tyrosine kinase inhibitor, and omeprazole induce *CYP1A1* gene expression through ligand-independent AHR activation [155–157]. Furthermore, in the absence of ligand, AHR activation is also affected by metabolic conversion of molecules into ligands or various compounds’ ability to affect other cellular pathways. In lymphocytes, RORgt can also form a complex with AHR to modulate IL-22 expression upon AHR activation [158]. Through redox modification of AHR, 1-(4-Chlorophenyl)-benzo-2,5-quinone activates an AHR-dependent but ligand-independent signaling pathway, thereby promoting AHR's nuclear translocation and activation of the target gene, *CYP1A1* [159].

## AHR, intestinal immunity and inflammation

### AHR and intestinal immunity, friend, foe or both?

AHR is widely expressed in immune cells [28]. It regulates innate and adaptive immune cell development and function in the gut [61]. AHR is essential for maintaining the homeostasis of the intestinal epithelium and associated immune cells and for generating appropriate responses to epithelial injury and invading pathogens [160–165]. The maintenance of AHR-dependent intraepithelial lymphocytes (IELs) contributes to intestinal epithelial cell (IEC) homeostasis. IECs play a vital role in integrating extra intestinal and internal signals and coordinating the ensuing subsequent response [166, 167].

Is the relationship between AHR and intestinal immunity a friend, foe or both? Sometimes, AHR regulates intestinal immunity via ligands, thereby maintaining immune homeostasis [164]. Proper activation of AHR can promote intestinal immunity and reduce the occurrence of intestinal inflammation [168]. However, excessive activation of AHR can impair intestinal immunity and promote intestinal inflammation and even intestinal cancer [28]. The relationship between AHR and intestinal immunity is dynamic and depends on the activity of AHR, its ligands and the physiological state of the body [169]. Accordingly, for the purpose of mediating the desired treatment outcome while minimizing the inherent risk of prolonged receptor activity, further discussion is required to determine the ligand dose-dependent level of AHR signaling.

### Effects of AHR activity on intestinal immunity and inflammation

**Fig. 2:**
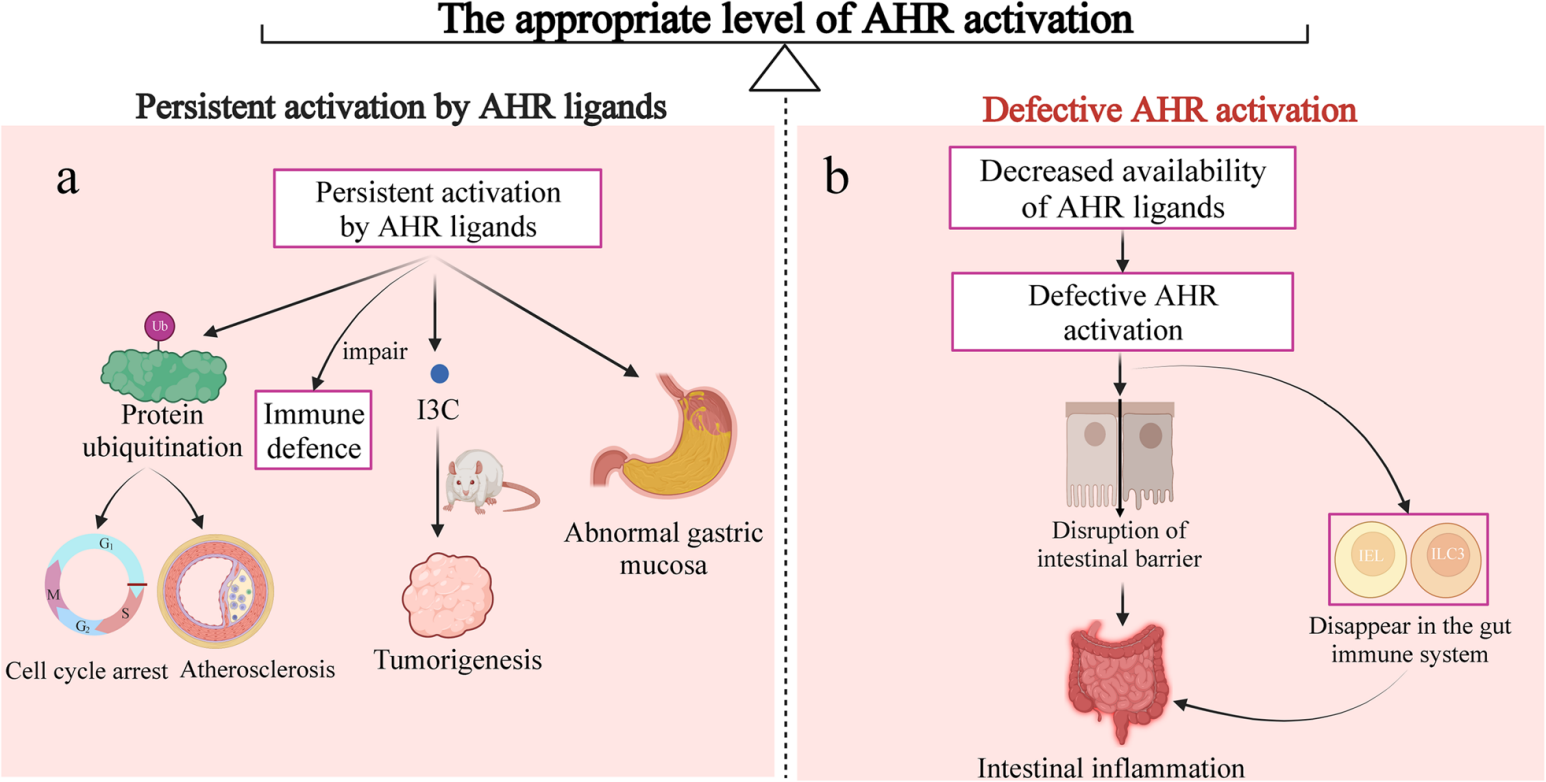
Different effects on intestinal immunity between persistent activation and defective AHR activation. **a**. persistent activation by AHR ligands has deleterious consequences. **b**. defective AHR activation is detrimental to the maintenance of intestinal homeostasis. This balance of intestinal homeostasis requires appropriate level of AHR activation. I3C, indole-3-carbinol; IEL, intraepithelial lymphocytes; ILC3, intraepithelial lymphocytes and type 3 innate lymphoid cells

Different levels of AHR activation generate distinct effects on intestinal immunity. Moderation is necessary to maintain homeostasis, and if the AHR activation level is lower or higher than optimal, intestinal disorders appear (Fig. 2).

Persistent activation by AHR ligands has a detrimental effect on intestinal health. Sustained TCDD-induced AHR activation usually results in rapid protein ubiquitination that targets the receptor for degradation by the proteasome [170–172], leading to toxic results such as cell cycle arrest, chloracne or increased atherosclerosis [2]. Sustained activation of AHR encourages tumor growth and affects immune defense [173]. Furthermore, excessive AHR signaling activation aggravates abnormalities in the gastric mucosa [174]. When compared to AHR homozygous mice, symptoms of colitis were dramatically ameliorated in AHR heterozygous mice, indicating that overstimulation of AHR may contribute to the development of colitis [175]. I3C, an AHR ligand, limits proinflammatory responses by suppressing nuclear factor kB signaling pathways [10]. In contrast, I3C administration can hasten the development of colonic lesions. Long-term in vivo treatment with I3C may be carcinogenic [176]. Negative feedback regulation is essential for AHR activation because prolonged stimulation is harmful. Excessive AHR activation can result in intestinal immune dysregulation and even intestinal diseases.

However, defective AHR activation is also detrimental to the maintenance of intestinal homeostasis. In the gut immune system, AHR deficiency in hematopoietic cells leads to IELs and type 3 innate lymphoid cells (ILC3) vanish [162, 177–179]. In the intestine, reduced AHR ligand availability results in defective AHR activation, which disrupts the intestinal barrier, alters immune responses and amplifies dysbiosis in chronic inflammation and consequently leads to IBD [167]. AHR ligand degradation has a detrimental impact on intestinal immune functions, which can be counterbalanced by increasing dietary intake of AHR ligands [180]. Therefore, an appropriate level of AHR activation is important to maintain intestinal homeostasis and maintain the intestine in a healthy state.

**Fig. 3:**
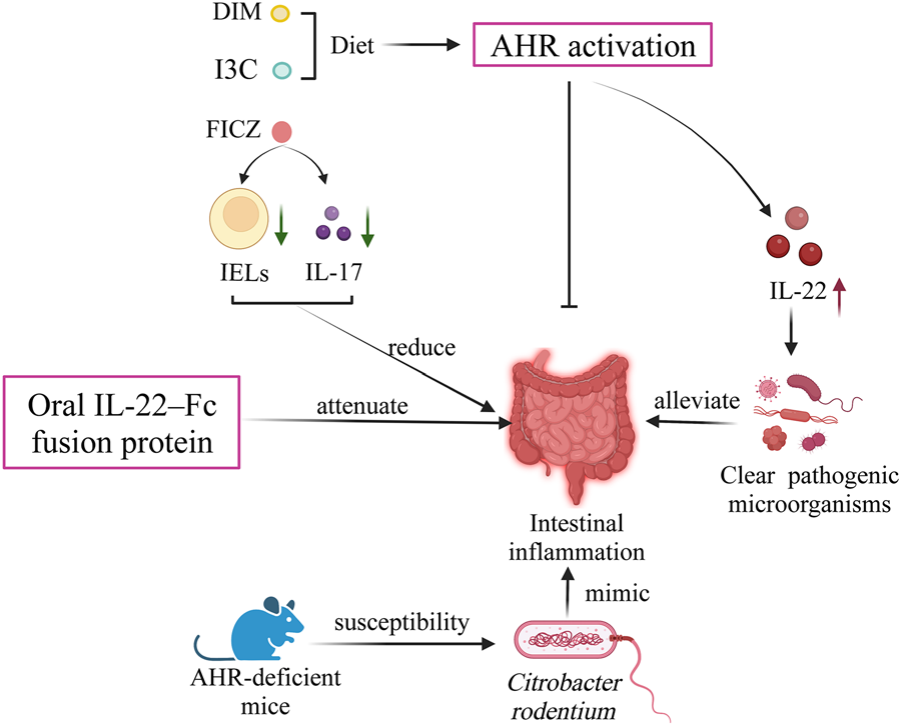
AHR activation could inhibit the intestinal inflammatory response. I3C, indole-3-carbinol; DIM, 3, 3-diindolylmethane; FICZ, 6-formylindoleo[3,2-b] carbazole; CYP1A1, cytochrome P450 1A1; IELs, intraepithelial lymphocytes; AHR, aryl hydrocarbon receptor

In terms of affecting intestinal inflammation, AHR activation could significantly inhibit the intestinal inflammatory response and alleviate the symptoms of colitis in experimental animals [161, 181, 182] (Fig. 3). AHR is a susceptibility locus for intestinal inflammation. Compared to healthy ones, AHR expression was decreased in the inflammatory tissues of Crohn’s disease patients, and the amount of natural AHR ligands was significantly lower in the feces of ulcerative colitis patients [183]. AHR activation regulates IEL and anti-inflammatory factors for the future treatment of intestinal inflammation. The AHR ligand FICZ ameliorates experimental colitis by lowering the number of activated IELs and reducing IL-7 production [28]. Additionally, AHR ligands or ligand precursors found in foods may prevent chemically induced intestinal inflammation. I3C or DIM supplementation reduces DSS-mediated colonic inflammation and disease severity [184]. AHR activation and the subsequent production of anti-inflammatory factors are important for the clearance of intestinal pathogenic microorganisms [180, 185]. Knockout studies have shown that AHR is indispensable for the treatment of intestinal inflammation. *Citrobacter rodentium* is commonly used to mimic the intestinal inflammation arising from enteropathogenic and enterohemorrhagic *E. coli* [186]. AHR-deficient mice exhibit increased sensitivity to *Citrobacter rodentium* due to their inability to produce IL-22 [187]. Higher sensitivity to *Citrobacter rodentium* was also observed in R26^Cyp1a1^ mice [180].

### Possible mechanisms of AHR effects on Intestinal Immunity and Inflammation

**Fig. 4:**
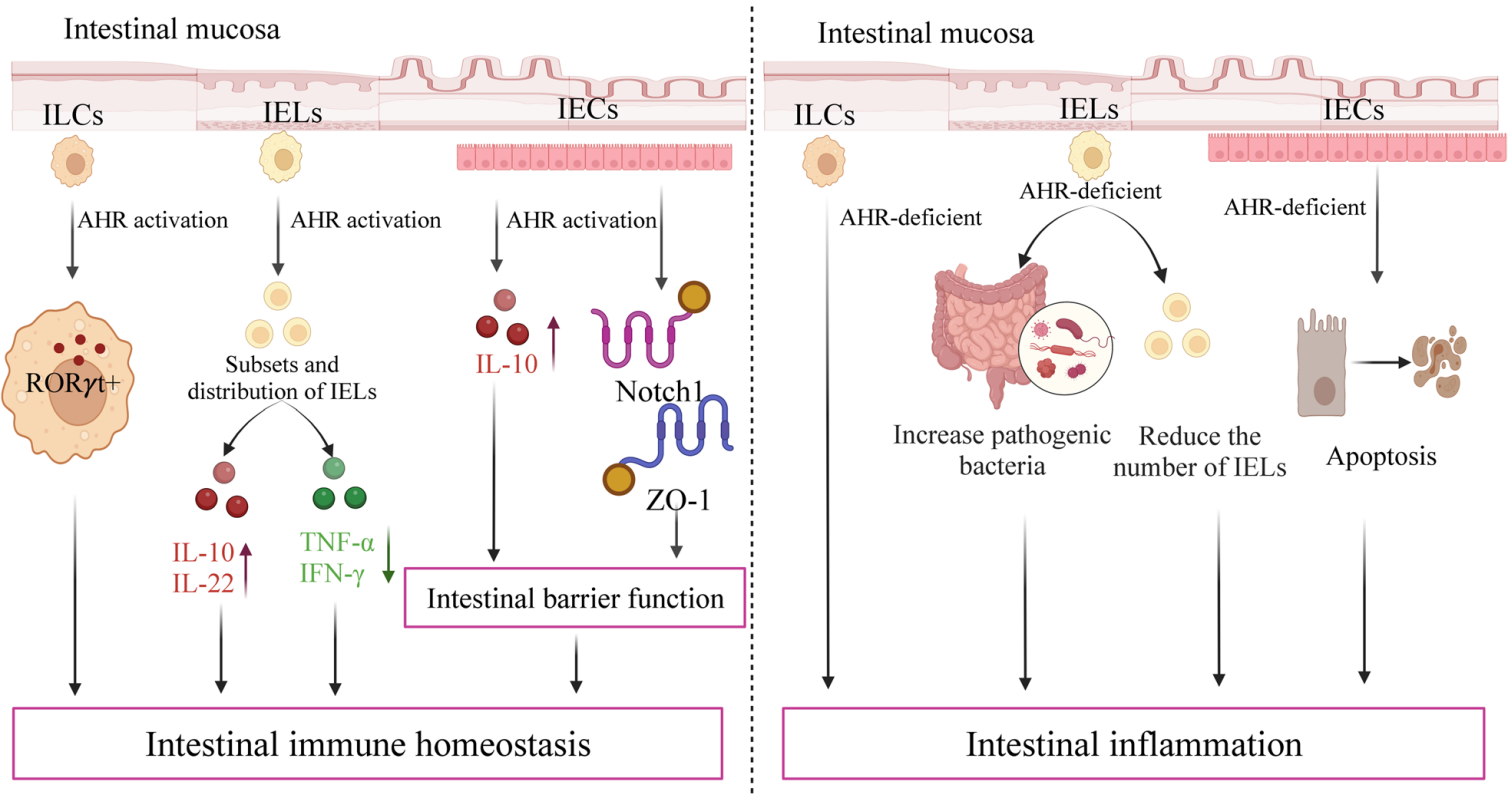
Schematic represents the effects of AHR on gut immunity and inflammation in intestinal mucosal immune system. Activation of AHR plays an important role in regulating intestinal immunity (on left). the deficiency of AHR results in intestinal inflammation (on right). ILCs, innate lymphoid cells; IELs, intraepithelial lymphocytes; IECs, intestinal epithelial cells; AHR, aryl hydrocarbon receptor; ROR γt + , RORγt + ILCs; Notch1, tight junction protein Notch1; ZO-1, tight junction proteins zonula occludens-1

**Fig. 5:**
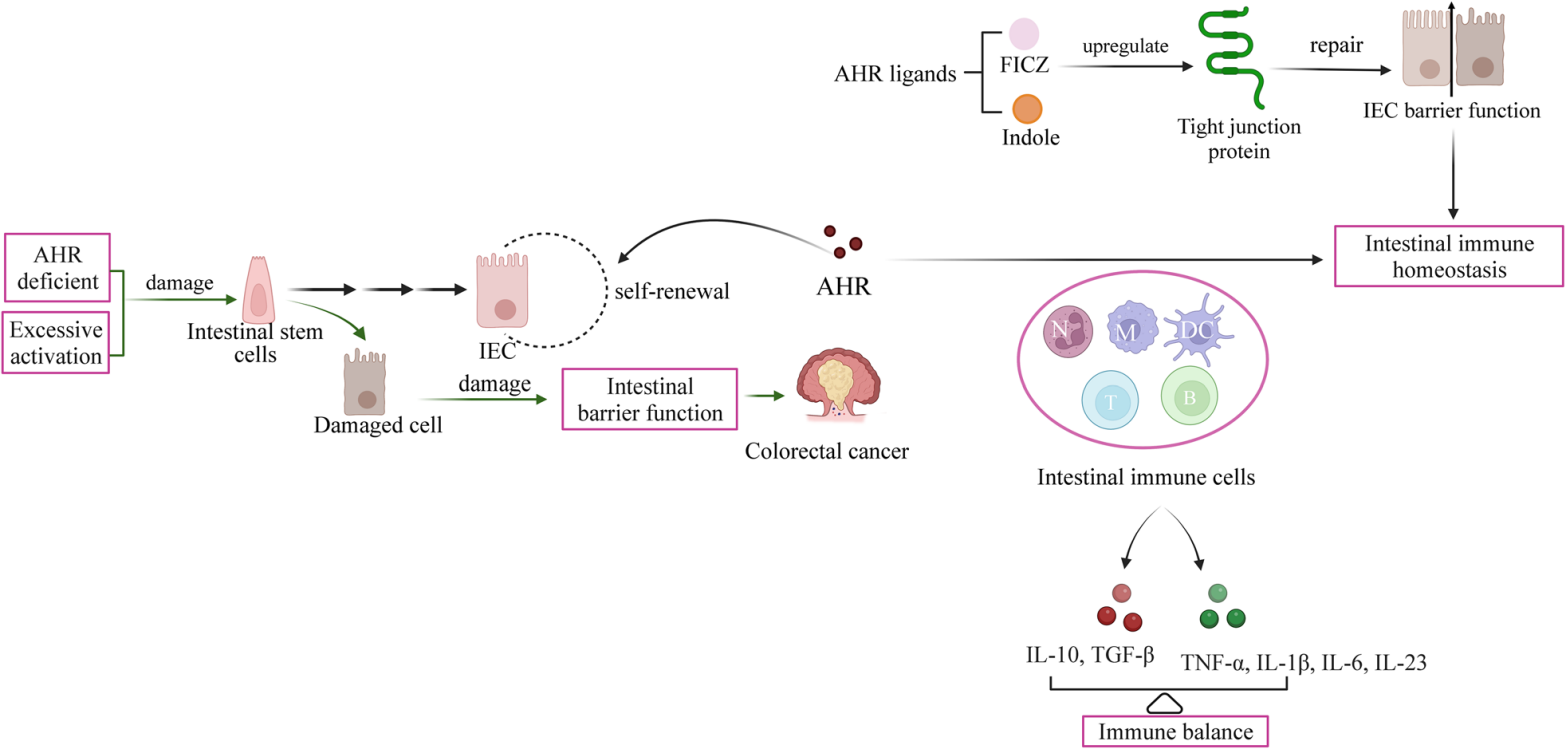
AHR affects intestinal immunity in two main ways: (1) AHR acts directly on intestinal epithelial cells (IECs). (2) AHR affects intestinal immunity through regulating intestinal immune cells. N, neutrophils; M, macrophages; DC, dendritic cells; T, T cells; B, B cells; IECs, intestinal epithelial cells; FICZ, 6-formylindoleo[3,2-b] carbazole; AHR, aryl hydrocarbon receptor

AHR is required for the regulation of intestinal immunity and inflammation (Fig. 4). The first physical barrier against pathogens and poisons entering the body through the digestive system is the intestinal mucosal immune system. It is primarily composed of innate lymphoid cells (ILCs), IELs and IECs [41]. The widespread expression of AHR in IELs and ILCs reveals its important function in the regulation of the intestinal immune system. Activation of AHR by ligands from dietary sources is crucial for regulating intestinal mucosal immunity and intestinal barrier function [181, 188]. AHR affects intestinal immunity in two main ways (Fig. 5): (1) AHR acts directly on IECs. AHR is necessary for the development and self-renewal of IECs derived from local stem cells [189, 190]. AHR is activated in IECs to enhance intestinal barrier function, alleviate inflammation, and maintain overall mucosal homeostasis [191, 192]. In addition, AHR slowed down the repositioning of zonula occludens-1 (ZO-1), increased the integrity of the intestinal epithelium, ameliorated hypoxia-induced changes in intestinal permeability, and maintained a normal intestinal barrier [188, 193]. Deficiency of AHR in IECs hampered the ability of intestinal stem cells to restore and differentiate in response to cell injury, having a significant impact on infection resistance and colorectal cancer development [194]. Furthermore, in the intestinal epithelium, AHR deficiency enhanced IEC apoptosis, impaired the proliferation of colonic crypt stem cells, and made mice more sensitive to DSS-induced intestinal inflammation [195, 196].

(2) AHR affects intestinal immunity by regulating intestinal immune cells. Intestinal immune cells mainly include IELs, Th17 cells, ILCs, neutrophils, macrophages, and dendritic cells [197–199]. These immune cells secrete various cytokines that regulate gut immune homeostasis, including proinflammatory factors (IL-1β, IL-6, IL-23, TNF-α) and anti-inflammatory factors (TGF-β, IL-10) [200, 201]. The balance of proinflammatory and anti-inflammatory factors is critical in preventing intestinal inflammatory diseases. In addition, it was shown that AHR affects intestinal immunity via interactions with numerous intestinal immune cells and cytokines [167, 180].

IELs are essential for intestinal immune development and intestinal inflammation. AHR is highly expressed on the IEL surface and influences the maintenance and differentiation of IELs [202, 203]. Also, AHR is an essential regulator for maintaining the number of IEL in the intestine. AHR activation can influence the subsets and distribution of IELs, which induce the upregulation of the anti-inflammatory factors IL-10 and IL-22 and the downregulation of the proinflammatory factors IFN-γ and TNF-α [181, 182, 204]. Instead, when the AHR is absent, the number of IELs is decreased, and the burden of intestinal bacteria is increased, which promotes dextran sulfate sodium (DSS)-induced colitis [205]. Collectively, AHR controls the number and homeostasis of IELs and further regulates intestinal immunity.

ILCs mediate the regulatory properties of AHR on the intestinal immune system [206]. ILCs are essential for the development of gut lymphoid follicles, epithelial homeostasis, defense against intestinal pathogens, and protection from inflammatory disorders [207, 208]. AHR is expressed in RORγt^+^ ILCs, and AHR signaling is considered necessary for these cells to expand and maintain homeostasis in the intestine [162]. ILCs and CD4 + T cells produce IL-22, which protect against intestinal inflammation [209]. IL-22 promotes IEC regeneration by increasing the production of anti-microbial peptides and mucins and enhancing the integrity of the mucosal barrier [210]. However, in mice exposed to DSS, the absence of IL-22 resulted in more severe colitis [211–213]. Thus, ILCs and IL-22 maintain the balance of intestinal immune homeostasis and protect against inflammation by modulating AHR.

Th17 cells and Tregs are both derived from CD4 + T cells [214]. AHR is highly expressed in Th17 cells and weakly expressed in Foxp3 + Treg cells, and its activation by FICZ enhances Th17 cell differentiation and promotes IL-22 production [44, 215]. In addition, AHR regulates Treg and TH17 cell differentiation in a ligand-specific manner [216]. I3C and DIM induce Tregs while suppressing Th17 cells, whereas FICZ has the opposite effect [217]. AHR regulates Tr1 cell metabolism and decreases HIF1α cellular levels, participating in the late stages of Tr1 cell differentiation [218]. AHR may be important in modulating TH17 cell function by inhibiting TH17 cell conversion to a TH1 cell-like profile and instead promoting TH17 cell transition to a Tr1 cell-like state [219]. At early stages of Th17 cell differentiation, AHR activation might convert Th17 cells into IL-10-producing immunosuppressive Tr1 cells [219, 220]. Tregs suppress inflammation and maintain immune tolerance by secreting IL-4, IL-10, and TGF-β [221]. Baicalein and DIM regulates Th17/Treg differentiation via AHR activation, thereby protecting against DSS-induced colitis in mice [222, 223]. Collectively, modulation of Th17/Treg homeostasis by ligand-activated AHR can improve intestinal immunity and relieve inflammation.

Th17 cells secrete interleukin-7 (IL-7), which is essential for T and B cell development as well as IEL differentiation and maturation [224–227]. Furthermore, IL-7, a novel target of the AHR pathway in the intestine, is a critical cytokine for triggering mucosal inflammation in IBD [203]. In DSS-induced colitis mice, AHR activation downregulates IL-7 and reduces inflammation [228]. Specifically, FICZ, as an endogenous ligand of AHR, reduces epithelial cell-derived IL-7 expression, concomitant with the amelioration of experimental colitis by reducing the frequency of activated IELs [181]. In short, IL-7 blockade mediates the favorable impacts of AHR pathway activation on colitis.

AHR is a key cofactor involved in IL-10 production by NK cells [229]. IL-10 can also be secreted T cells, B cells, macrophages, dendritic cells, eosinophils and neutrophils [230]. As an anti-inflammatory cytokine, IL-10 is essential for maintaining gut immune homeostasis and the intestinal mucosal barrier [231]. The AHR-Src-STAT3 pathway is required for inflammatory macrophages to produce IL-10 [232]. In addition, kynurenine activates AHR, resulting in the upregulation of IL-10R1 in IECs and reducing mucosal inflammation [233, 234]. In brief, immune cells and cytokines provide a regulatory immunological and inflammatory response via AHR activation.

## AHR, a potential target for maintaining intestinal health

### AHR, a potential target to maintain intestinal homeostasis

Since AHR plays a vital role in intestinal health, it is feasible to improve intestinal homeostasis by regulating this potential target. AHR promotes immune homeostasis through a variety of mechanisms, including T-cell differentiation and Th17 development, as well as increased IL-22 production [161, 218, 235]. After binding to a ligand, the AHR can interact with DREs in the promoter regions of IL-10 or IL-22, promoting their production in gut ILCs, DCs, and Treg cells [236, 237]. Elevated IL-10 can promote the generation of tolerogenic DCs and Treg cells while inhibiting Th17 cell differentiation, resulting in a decrease in proinflammatory cytokines that mediate gut microbial composition and host homeostasis [238].

The microbiota has a crucial function in gut homeostasis. Microbial dysbiosis, which results in altered L-tryptophan metabolism, decreased AHR activation, and insufficient IL-22 levels, can promote a vicious cycle that promotes gastrointestinal homeostasis loss. Supplemental IL-22 may reverse this process, remodeling the microbiome to increase AHR activity and enhance a virtuous cycle to help regain homeostasis [239]. The intestinal microflora produces tryptophan/indole metabolites that act as AHR ligands. Indole metabolites enhance intestinal homeostasis through AHR-mediated regulation of the IEC IL-10R1 [110]. Indoles, as AHR ligands, have anti-inflammatory activities, maintaining intestinal homeostasis [240]. *Lactobacillus spp.* can convert tryptophan to IAld, which are AHR ligands. They can activate AHR and further encourage intestinal homeostasis by inducing IL-22 [20]. The SCFA butyrate as an AHR ligand, which is produced by the intestinal flora, contributes to the maintenance of intestinal immune homeostasis via encouraging the differentiation of IL-10-producing Treg and Tr1 cells [241–243]. Therefore, the intake of these AHR ligands may be a strategy for maintaining intestinal health.

Under normal conditions, persistent organic pollutants alter gut microbiota-host metabolic homeostasis via AHR overactivation [22]. Sustained potent activation by exogenous synthetic AHR ligands may cause intestinal dysfunction. After 5 days of exposure to the strong and long-lasting xenobiotic pollutant 2,3,7,8-tetrachlorodibenzofuran, mice developed microbial imbalance and disease [22]. Furthermore, high-dose PCB126 and B(a)P exposure adversely affected the microbiota community structure [244, 245]. Hence, minimizing the intake of exogenous synthetic AHR ligands is important to prevent intestinal dysfunction caused by overactivation of AHR.

The consumption of AHR ligands is crucial for the effects of AHR on intestinal homeostasis [246]. Appropriate levels of AHR activated by some exogenous ligands (vegetables or other beneficial ligands) can effectively maintain intestinal homeostasis. I3C and DIM maintain the number of lymphocytes in the intestinal epithelium and protect the intestinal mucosal barrier [205]. A lower level of AHR activation by endogenous ligands facilitates the maintenance of intestinal homeostasis [167].

Moderate AHR activity is essential for maintaining proper intestinal immune homeostasis under normal conditions. Accordingly, the intake of beneficial dietary ligands should be increased, and the intake of harmful ligands should be reduced, which can enhance epithelial barrier function, protect against intestinal challenges.

### A potential therapeutic target in intestinal inflammation

Studies have proven a positive correlation between the degree of inflammation and the level of AHR activation [247]. Dysregulated AHR activity adversely affect intestinal infection and inflammation through intestinal intraepithelial lymphocytes loss [248]. AHR ligands containing xenobiotics (TCDD), endogenous substances (FICZ, norisoboldine) and dietary products (soy isoflavones, arachidonic acid, quercetin and baicalein) can activate AHR, suppress inflammatory responses and alleviate the symptoms of colitis [161, 249, 250]. TCDD ameliorates the symptoms of colitis by inhibiting Th17 cell differentiation and decreasing the expression of IL-17 and IFN-γ [251]. FICZ, a tryptophan photoproduct, can alleviate both DSS-induced enterocolitis and trinitrobenzene sulfonic acid or T-cell transfer-induced colitis by reducing the production of proinflammatory cytokines and increasing the production of anti-inflammatory IL-22 by Th17 cells [175, 204, 252]. Norisoboldine stimulates Treg differentiation and suppresses the NLRP3 inflammasome to alleviate the symptoms of colitis [253]. In mouse models with gastrointestinal inflammation, AHR ligand provision can directly attenuate inflammatory signaling [254]. When Qing-Dai was administered, the inflammatory responses of colonic macrophages as well as the generation of TNF-α, IL-1β, and IL-6 in colonic tissue were suppressed [255]. The AHR agonist β-naphthoflavone was administered orally and reduced DSS-induced colitis [256]. I3C produced from vegetables was administered to suppress the development of acute colitis brought on by DSS [177]. When glucosinolate-rich cabbage was consumed, the AHR target gene *CYP1A1* was substantially upregulated in the colon, which also reduced colitis [257]. Mice lacking AHR had more severe colitis, whereas those treated with AHR agonists had attenuated disease progression [28]. Kurarinone (KAR) has a therapeutic role in irritable bowel syndrome (IBS) by modulating macrophage functions via stimulating AHR signaling. When AHR deficiency in macrophages, the effect of KAR in IBS mice was weakened [258].

Some bacteria have immunoregulatory effects in the gut by activating the AHR pathway, which culminates in anti-inflammatory responses [259, 260]. A probiotic bacterium can control intestinal inflammation via AHR in the presence of tryptophan [28]. *Lactobacillus bulgaricus* strain OLL1181 alleviated DSS-colitis by activating AHR signaling and increasing the expression of *CYP1A1* [261]. Oral administration of 1,4-dihydroxy-2-naphthoic acid (DHNA), an AHR activator derived from the cheese bacteria *Propionibacterium freudenreichii* ET-3, induced anti-microbial peptides in the intestine, further controlling inflammation in DSS-colitis [262].

Thus, AHR has the potential as a drug target for the treatment of colitis. Furthermore, the intestinal environment is extremely complex and uncertain. The variables affecting the production and regulation of AHR ligands are numerous and complex, including changes in diet and gut microbes. Hence, the impact of AHR ligands on intestinal immunity in this complex environment should be considered before treating intestinal diseases. More research is needed to accurately evaluate ligands and activation effects in the complex intestinal environment.

## Dietary habits affect intestinal health through AHR

Dietary habits can directly affect intestinal immunity. Vegetable, tryptophan and microbial metabolism, SCFA, and natural plant extracts are the largest sources of AHR ligands [241] (Table 2). Dietary AHR ligands have a transient effect on intestinal health that varies greatly depending on the ligand types, bioavailability, and half-life.

**Table 2:** A list of AHR Ligand abundant in diet

Dietary	AHR ligand	References
*Brassica* vegetables	Indolyl glucosinolates	Li et al. 2011 [205]
	I3C	Bjeldanes et al. 1991; Hammerschmidt-Kamper et al. 2017; Ito et al. 2007 [80, 355, 356]
	LTr-1	Bjeldanes et al. 1991[80];
	DIM	Bjeldanes et al. 1991; Yin et al. 2012 [80, 357]
	ICZ	Bjeldanes et al. 1991; Jellinck et al. 1993 [80, 358]
Tryptophan and microbial metabolism	IA	Vyhlídalová. et al. 2020 [359]
	IAA	Miller 1997; Jin et al. 2014 [360, 361]
	IPA	Aoki R et al. 2018 [354]
	IAld	Bjeldanes et al. 1991; Zelanteet al. 2013 Alexeev et al. 2018[20, 80, 110]
	IAAld	Agus et al. 2018 [362]
	ILA	Meng et al. 2020 [363]
	Skatole	Weems and Yost et al. 2010; Hubbard 2015 [113, 364]
SCFA	Butyrate	Marinelli et al. 2019 [365]
Natural plant extracts	Berberin	Jing et al. 2021 [366]
	Resveratrol	Casper et al. 1999 [367]
	Mangosteen ketones	Tocmo et al. 2020 [368]
	Flavonoids	Zhang et al. 2003 [79]
	Indigo naturalis	Adach et al. 2001 [118]

### Vegetable intake and associated AHR ligands

**Fig. 6:**
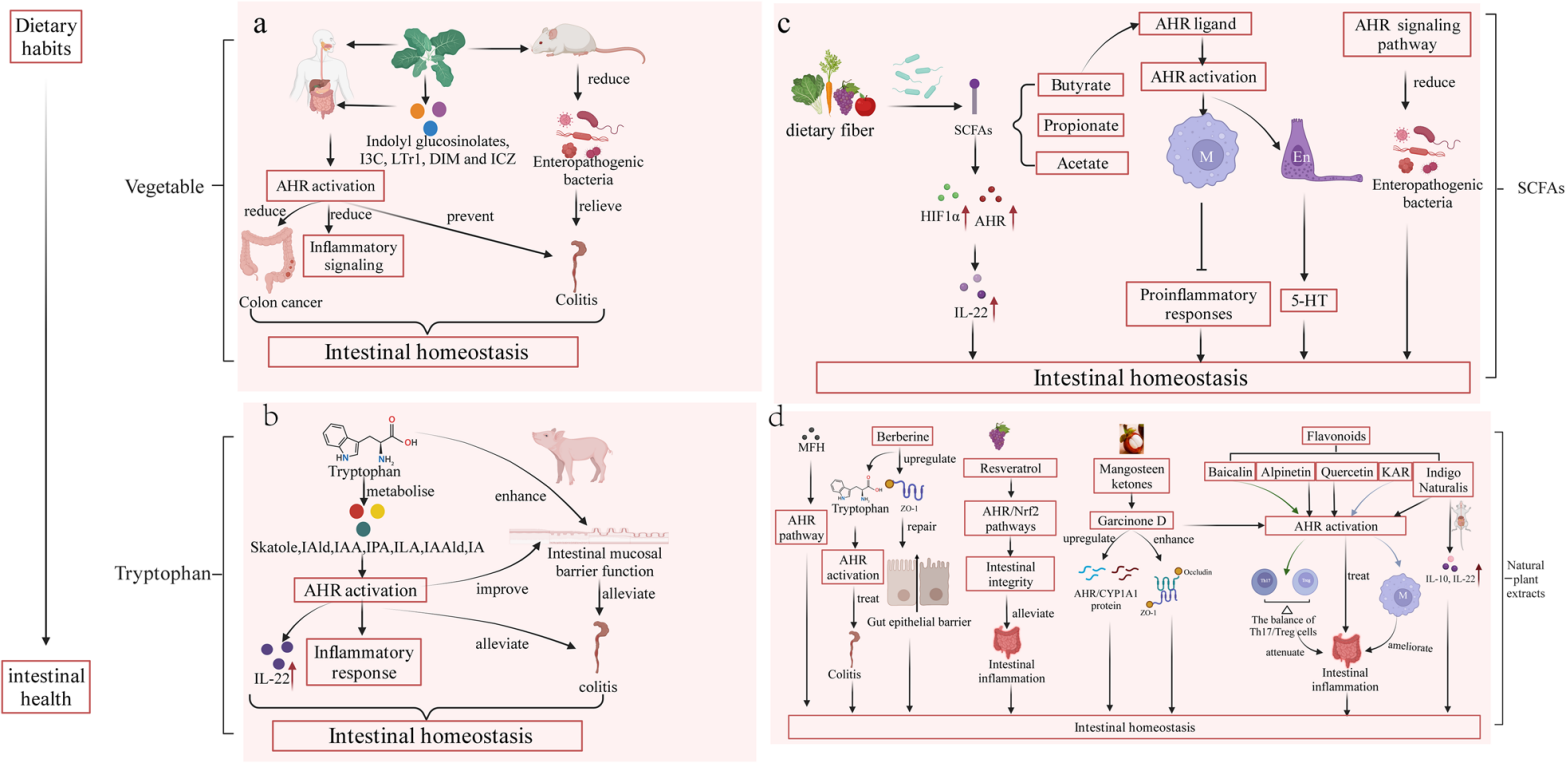
Dietary habits affect intestinal health through AHR. **a** Consumption of *Brassica* vegetables lead to the formation of AHR ligands, which affect intestinal health. **b** Tryptophan affect intestinal health through AHR. **c** Dietary fibers influence intestinal health through AHR. **d** Natural plant extracts affect intestinal health through AHR. I3C, indole-3-carbinol; DIM,3,30- diindolylmethane; ICZ, indolo[3,2-b] carbazole; LTr-1, 2-(indole-3-methane)-3, 3'-diindolylmethane; IAld, indole-3-aldehyde; IAA, indole-3-acid-acetic; IPA, indole-3-propionic acid; ILA, indolelactic acid; IAAld, indole-3-acetaldehyde; IA, indoleacrylic acid; skatole, 3-methylindole; HIF1α, hypoxia- inducible factor 1α; MFH,medicine food homology; M, macrophage; En, enterochromaffin cells; Treg, regulatory T cell; Th17, T helper cell 17; SCFAs, Short-chain fatty acids; ZO-1, tight junction proteins zonula occludens-1; KAR, kurarinone

Consuming *Brassica* vegetables, which are known to promote AHR activation, is linked to decreased inflammatory signaling and a lower risk of colon cancer [257] (Fig. 6a). Several studies have reported that indolyl glucosinolates, I3C, LTr-1, DIM and ICZ, which are abundant in cruciferous vegetables, are AHR ligands [263]. I3C or glucobrassicin supplementation in rodents' diets can prevent DSS-induced colitis [177, 254, 264]. Additionally, the administration of broccoli could change the resident microflora and reduce intestinal inflammation [265]. Notably, the therapeutic effect of maintaining homeostasis in the gut after broccoli eating is mediated by AHR activation. Overall, consuming vegetables can enhance AHR signaling and maintain intestinal barrier homeostasis, which is beneficial to gut health. As a result, selecting vegetable cultivars with higher levels of glucosinolates, which produce AHR ligands, may have greater health benefits.

### Tryptophan and microbial metabolism

AHR is essential for linking tryptophan catabolism between microbial communities and hosts (Fig. 6b). Dietary tryptophan can improve the function of the intestinal mucosal barrier, alleviate acute colitis, and maintain epithelial homeostasis via AHR [115, 266]. Intestinal microorganisms directly convert tryptophan to indole and its derivatives. Many indole derivatives, including IAld, IAA, IPA, ILA, IAAld and IA, are AHR ligands [110]. Likewise, tryptamine and skatole also act as ligands of AHR.

Recently, several studies have demonstrated that intestinal microorganisms produce various tryptophan catabolites. For instance, *Clostridium sporogenes, Peptostreptococcus spp*. and *Lactobacillus spp.* can convert tryptophan into tryptamine, ILA, IA, IAld and IPA [20, 114, 267–269]. Skatole, a common intestinal metabolite, is produced by decarboxylation of IAA from *Bacteroides spp.* and *Clostridium spp.* [270–273]. How do tryptophan metabolites affect intestinal health? Skatole induces the activation of AHR, thereby regulating IEC death [274]. IA can attenuate the inflammatory response and repair barrier function by promoting the formation of mucus and the differentiation of goblet cells by activating AHR [275, 276]. ILA protects cultured intestinal epithelial cells by activating the AHR and Nrf2 pathways [277]. In mice fed a high-fat diet, IPA reduces intestinal permeability [278]. Both oral indole and IPA can improve colonic inflammation in mice [279, 280]. AHR activation by IAld induces IL-22, thereby maintaining intestinal homeostasis [20]. Tryptophan can be metabolized to numerous AHR ligands via a variety of metabolic pathways. These AHR ligands can activate AHR and the expression of downstream target genes like IL-22 and IL-17, which are beneficial to maintaining intestinal homeostasis.

### SCFAs

SCFAs are derived from the bacterial metabolism of ingested fibers, including acetate, propionate, and butyrate [281]. SCFAs regulate the intestinal immune system in a variety of ways by inducing and regulating T cells and constraining cytokine responses [242, 282–284] (Fig. 6c).

In the intestine, SCFAs regulate AHR and its target genes [285]. Among the SCFAs, butyrate, as a ligand, activates AHR in human IECs and inhibits proinflammatory responses by intestinal macrophages [286]. The butyrate-serotonin-AHR axis influences intestinal immune homeostasis. Butyrate via AHR activation promotes 5-HT release from neural enterochromaffin cells to regulate intestinal homeostasis and peristalsis [287]. 5-HT induces the expression of *CYP1A1* in IECs via AHR activation [288]. In addition, the AHR signaling pathway can affect the composition of small intestinal flora, thus regulating intestinal flora balance and maintaining intestinal health [24].

Dietary habits influence SCFA levels in the intestine. A Mediterranean diet high in fruits, vegetables, and legumes is linked to an increase in fecal SCFA levels [289, 290]. A high-fiber diet led to an obvious rise in gut SCFAs [291]. In addition, SCFAs upregulate IL-22 production by encouraging the expression of AHR and hypoxia-inducible factor 1α to maintain intestinal homeostasis [292]. Fermentable dietary fibers influence the production of SCFAs, and SCFAs exert important regulatory functions on intestinal health by regulating the AHR pathway. Due to this, the Mediterranean diet and fermentable dietary fiber may maintain intestinal homeostasis partially via modulation of AHR.

### Natural plant extracts

Natural plant extracts, including berberine, resveratrol, mangosteen ketones, flavonoids, indigo naturalis and more, can treat intestinal inflammation to regulate intestinal immunity through AHR. Among these, berberine, baicalin, alpinetin, quercetin and indigo are all AHR ligands (Fig. 6d).

Berberine, a protoberberine alkaloid from several plant species, is an AHR ligand [293, 294]. Berberine has significant therapeutic potential against IBD. It may reduce gut epithelial barrier dysfunction via increasing the expression of ZO-1 and treat DSS-induced colitis by modulating the activation of AHR by tryptophan metabolites associated with the intestinal microbiota [297–300]. Resveratrol, a known AHR antagonist, is a natural polyphenol found in grape skin and red wine [301]. In weaned pigs exposed to diquat, resveratrol modulates the AHR/Nrf2 pathways to lessen intestinal inflammation and protect intestinal integrity [88].

Mangosteen ketones from the tropical fruit mangosteen (*Garcinia mangostana*) showed anti-inflammatory and antioxidant activities. Garcinone D, one of the seven acylated xanthones contained in mangosteen, can activate AHR, significantly upregulate AHR/CYP1A1 protein expression and enhance ZO-1 and occludin protein levels. Additionally, Garcinone D prevented intestinal epithelial barrier dysfunction brought on by oxidative stress [302].

Flavonoids have anti-inflammatory and chemopreventive properties [303, 304]. Specific examples of flavonoids include baicalin, alpinetin, quercetin and KAR. Baicalin, isolated from *Radix scutellariae*, is a novel AHR ligand that restores the balance of Th17/Treg cells via AHR to alleviate colitis [222]. Baicalein strengthens the intestinal epithelial barrier through the AHR/IL-22 pathway in ILC3s, thereby reducing the symptoms of ulcerative colitis [305]. As a potential AHR activator, alpinetin regulates Treg differentiation, thereby reducing the symptoms of colitis [306–308]. Quercetin, a clear AHR agonist, could shorten the course of chronic DSS colitis in an AHR-dependent manner [309] KAR, a flavonoid derived from *Sophora flavescens*, is an effective treatment of visceral hypersensitivity in IBS, and it regulates the development of IBS through macrophage-intrinsic AHR [258].

Indigo Naturalis is derived from indigoferous plants [310, 311]. It has been demonstrated that Indigo, one of the main components of Indigo Naturalis, is an AHR ligand. Indigo alleviated murine colitis by activating AHR signaling [118, 195, 312]. Indigo promotes colon epithelial cell proliferation and migration by activating AHR in intestinal epithelia, thereby exerting its erosion-healing effects [313]. Moreover, adding Indigo Naturalis to DSS-induced colitis mice can promote the expression of IL-10 and IL-22 in colonic lamina propria lymphocytes but not in AHR-deficient mice, which may be related to reduced production of regulatory cytokines [314].

The components of medicine food homology (MFH) are complex and can treat intestinal diseases by exerting anti-inflammatory and antioxidant effects, as well as regulating the intestinal flora [315]. In addition, a growing body of research has demonstrated that TCM has great potential in treating intestinal diseases by regulating intestinal immunity and inflammation through the AHR pathway [316]. Eating MFH will have a beneficial effect on intestinal health. IBD is relieved by *Hericium erinaceus*, a traditional edible mushroom. It regulates intestinal bacteria and the immune system [317]. The major traditional uses of berberine or berberine-containing plants have been for treating intestinal infections such as gastroenteritis, cholera, and dysentery. Extracts of barberry and Oregon grape are used for balancing the intestinal flora in the digestive tract [318]. Phytochemicals derived from natural products, including alpinetin, baicalin, curcumin, resveratrol, indirubin, berberine, and norisoboldine, are effective Th17/Treg regulators and have anti-inflammatory properties in the colon [319].

Both cardamonin and norisoboldine (NOR) from Tilia can alleviate TNBS-induced colitis in mice by activating AHR and eventually inhibiting the activation of colonic NLRP3 inflammatory vesicles [250, 320]. The anti-inflammatory effect of Magnolol on DSS-induced colitis in mice was primarily due to the restoration of colitis serum tryptophan metabolites KA, 5-HIAA, IAA, and indoxylsulfuric acid, all of which are AHR ligands [321]. Galangal ameliorated colitis in mice by activating AHR, upregulating *Foxp3* expression, and restoring Th17/Treg balance [322]. Naringenin activates AHR, causing naive T cells to differentiate into Treg cells while inhibiting differentiation into Th17 and Th1 cells, increasing the ratio of Treg cells in the peripheral blood of mice with colitis and thus alleviating colitis [323, 324]. In addition, Qing Dai and indigo may improve colitis by activating AHR to upregulate IL-10 and IL-22 [325].

Altogether, berberine, resveratrol, mangosteen ketones, flavonoids and indigo naturalis promote intestinal self-healing by activating the AHR, lowering intestinal inflammation, altering the ecology of intestinal microorganisms, and mediating immunomodulation. Additionally, natural plant extracts regulate intestinal flora and organismal metabolism. The effect of microbial metabolites on AHR requires further elucidation, which may guide the development of AHR-targeted immunomodulators, further benefiting the healthy development of the intestinal tract.

## Conclusion and perspectives

AHR regulates different types of cells in the gut, including ILCs, IELs and IECs, and it is essential for maintaining intestinal immune homeostasis. In addition, the sources and structures of AHR ligands are diverse, and they act differently. AHR ligands are derived from diet, gut microbial metabolites and the environment. They are enriched in the gastrointestinal tract and activate AHR in the gut. Dietary AHR agonists, indole derivatives and/or probiotics that produce AHR ligands have significant potential for preventive and therapeutic interventions against intestinal inflammation. Under normal conditions, moderate activation of AHR regulates intestinal immune homeostasis. When the activation of AHR is excessive or insufficient, it can affect intestinal immune disorders and result in the formation of intestinal diseases and even cancer. Furthermore, the AHR pathway is a very promising therapeutic target for the regulation of intestinal inflammation, but there are still some scientific challenges to be addressed.

The side effects of numerous AHR ligands are challenges to the therapeutic effect of AHR. Many AHR agonists, such as FICZ and TCDD, have been shown to alleviate colitis symptoms [14, 127, 128]. Unfortunately, long-term use of these compounds causes severe side effects [2–5, 326, 327], limiting their use as therapeutic agents in animals or humans. New or existing AHR-targeting drugs with high efficiency and minimal side effects should be filtered for better therapeutic effect.

In addition, accurately evaluating ligand and activation effects in the complex intestinal environment also poses challenges for the therapeutic potential of AHR. Dietary and medicinal plant extracts have been shown to suppress inflammatory responses and alleviate the symptoms of colitis [99, 222, 305]. However, since the background content of AHR ligands is not low in the intestinal environment, activation of AHR signals itself cannot be dismissed. So, more research is needed to determine how to eliminate the influence of preexisting ligands in the intestine to evaluate the activational effect of a single ligand or assess the levels of background AHR activation before treatment with AHR ligands.

## Data Availability

Not applicable.

## Abbreviations

AHR: Aryl hydrocarbon receptor
TCDD: 2,3,7,8-Tetrachlorodibenzo-p-dioxin
I3C: Indole-3-carbinol
DIM: 3, 3-Diindolylmethane
ICZ: Indolo[3,2-b] carbazole
FICZ: 6-Formylindoleo[3,2-b] carbazole
dFICZ: 6,12-Diformylindolo[3,2-b] carbazole
KYN: Kynurenine
SCFAs: Short-chain fatty acids
IAld: Indole-3-aldehyde
HAHs: Halogenated aromatic hydrocarbons
PAHs: Polycyclic aromatic hydrocarbons
ARNT: AHR nuclear transport
DC: Dendritic cells
BaP: Benzo(a)pyrene
BA: Benz(a)anthracene
LTr-1: 2-(Indole-3-methane)-3, 3'-diindolylmethane
CA: Cinnabarinic acid
KA: Kynurenic acid
XA: Xanthurenic acid
ITE: 2-(10H-indole-30-carbonyl)-thiazole-4carboxylic acid methyl ester
3-IAld: Indole-3-carboxaldehyde
skatole: 3-Methylindole
IAA: Indole-3-acid-acetic
IPA: Indole-3-propionic acid
IAAld: Indole-3-acetaldehyde
IA: Indoleacrylic acid
ILA: Indolelactic acid
IELs: Intraepithelial lymphocytes
IECs: Intestinal epithelial cells
ILCs: Innate lymphoid cells
IL-7: Interleukin-7
*CYP1A1*: Cytochrome P450 1A1
*CYP1B1*: Cytochrome P450 1B1
I3ACN: Indole-3-acetonitrile
PCBs: Polychlorinated biphenyls
LXA4: Lipoxin A4
ILC3: Intraepithelial lymphocytes and type 3 innate lymphoid cells
KAR: Kurarinone
IBS: Irritable bowel syndrome
APC: Adenomatous polyposis coli
ZO-1: Zonula occludens-1
TCM: Traditional Chinese medicines
MFH: Medicine food homology
